# High Density Lipoproteins: Metabolism, Function, and Therapeutic Potential

**DOI:** 10.3389/fcvm.2020.00039

**Published:** 2020-03-31

**Authors:** Anne Jomard, Elena Osto

**Affiliations:** ^1^Laboratory of Translational Nutrition Biology, Swiss Federal Institute of Technology (ETH), Zurich, Switzerland; ^2^Institute of Clinical Chemistry, University Hospital Zurich, Zurich, Switzerland; ^3^Department of Cardiology, Heart Center, University Hospital Zurich, Zurich, Switzerland

**Keywords:** high density lipoprotein, cardiovascular risk, obesity, endothelial function, HDL-therapy, bariatric surgery, lipoproteins

## Abstract

High Density Lipoproteins (HDLs) have long been considered as “good cholesterol,” beneficial to the whole body and, in particular, to cardio-vascular health. However, HDLs are complex particles that undergoes dynamic remodeling through interactions with various enzymes and tissues throughout their life cycle, making the complete understanding of its functions and roles more complicated than initially expected. In this review, we explore the novel understanding of HDLs' behavior in health and disease as a multifaceted class of lipoprotein, with different size subclasses, molecular composition, receptor interactions, and functionality. Further, we report on emergent HDL-based therapeutics tested in small and larger scale clinical trials and their mixed successes.

## HDLs, Where are We Now?

### HDLs Historically: “Good Cholesterol”

**Figure 1:**
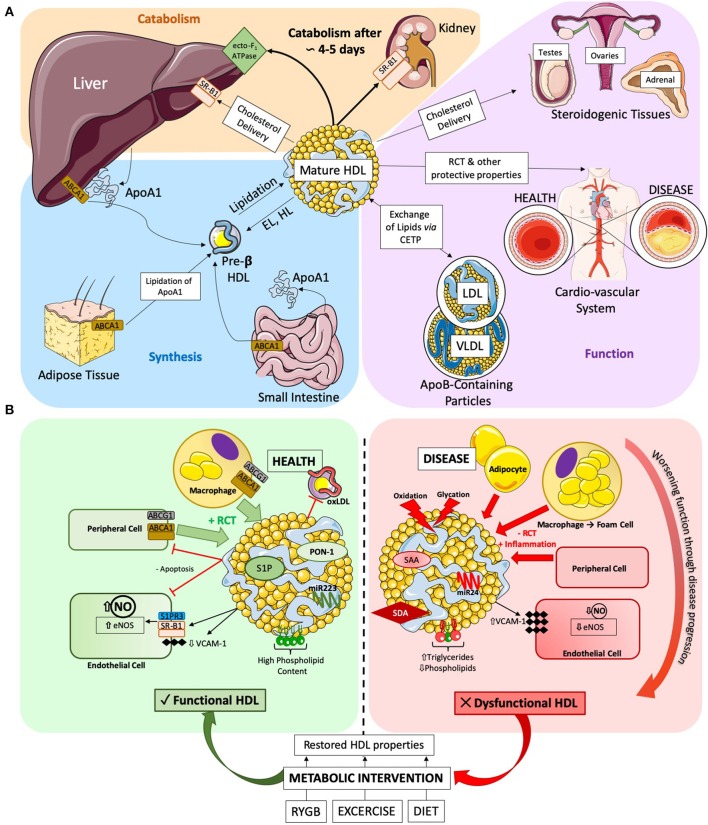
**(A)** HDL Lifecycle. Diagram detailing the three key stages of the HDL lifecycle. (1) Synthesis: ApoA1 is synthesized in the liver and the gut, where it can be gradually lipidated on-site or by the adipose tissue to produce pre-ß HDLs. Further lipidation results in mature HDL formation, which can in-turn become pre-ß HDL via the catabolic action of endothelial (EL) and hepatic (HL) lipases. (2) Function: HDLs main function are to efflux cholesterol and other lipids from peripheral tissues (such as the cardio-vascular system) and transport them either to (a) the liver for disposal, (b) steroidogenic tissues to support hormone production or (c) exchange lipids with apoB-containing particles. (3) Catabolism: finally, after a roughly 4 to 5 day lifecycle, HDLs are permanently catabolized either in the liver via the ecto-F_1_-ATPase or through complete delipidation by SR-B1 in the kidney and urinary excretion. **(B)** Diagram detailing the various actions of HDLs in health and disease. Healthy HDLs have a high PL content and are highly associated to beneficial molecules, such as S1P and PON-1 enzyme exerting a beneficial role on ECs, or anti-atherosclerotic miRNA 223. Throughout the pathogenesis of cardiovascular disease, HDLs becomes progressively more dysfunctional. The lipidome and proteome of HDLs are altered, with increased TG and decreased PL. SAA and SDA are become associated to HDL. Dysfunctional HDLs also present an altered miRNA profile, with increase in pro-inflammatory miRNA 24. Metabolic interventions have been shown to improve HDL functionality. RYGB, exercise, and diet restore HDL functionality and alter composition to varying degrees. SAA, Serum Amyloid A; SDA, Symmetric Dimethylarginine.

The term “good cholesterol” is often used with reference to the cholesterol content (HDL-cholesterol) in high-density lipoproteins (HDLs). The 1980's Framingham study found a strong positive association between coronary heart disease and low HDL-C levels (1). Thus, approaches were developed to increase HDL-C and achieve cardioprotection (2, 3). Notably, the ILLUMINATE Phase 3 trial, using the drug torcetrapib, increased HDL-C content significantly through inhibition of cholesteryl ester transfer protein (CETPi), which normally catalyzes the transfer of cholesterol from HDL to low density lipoproteins (LDL), and triglycerides from LDL to HDL (see Figure 1A). However, the trial was prematurely terminated as patients on torcetrapib showed higher risk of death and adverse cardiovascular events than the control group on atorvastatin (4). Other clinical trials testing different CETPi yielded similarly disappointing results, where increasing HDL-C resulted in no (5, 6) or marginal improvement in the cardiovascular end-points (7), either myocardial infarction or mortality (8). Anacetrapib was the only CETPi to show modest reduction of major cardiovascular events over a follow-up period of 4 years in the REVEAL trial (7), however the achieved benefits seem more attributable to the concomitant decrease in LDL-C, than to the HDL-C raising effects of the drug (9). Pharmacogenetic interactions driven by still unknown genetic variants in the population may have confounded the CETPi trial results, although there is no clear evidence to date (10). Beyond the CETPi trial results, the fact that HDL-C *per se* is not causally associated with cardiovascular benefits was supported by Mendelian randomization studies, demonstrating that genetic polymorphisms associated with increased HDL-C had no impact on the risk of myocardial infarction (11, 12). Evidence coming also from meta-analysis could not find an improvement in cardiovascular outcome after raising HDL-C levels (13). Interestingly, what was proven to be inversely associated with cardiovascular risk (14) was the pivotal biological function of HDL, known as reverse cholesterol transport (RCT), whereby HDL accepts excessive cholesterol from macrophages in peripheral tissues and carries it to the liver for disposal. Overall, these results ushered in a new era of research on HDL, focusing more on quality of whole HDL, rather than merely cholesterol content. HDLs are a family of particles that can exhibit fundamentally different metabolism and functions based on their specific proteomic, lipidomic, and physico-chemical properties. Further, HDLs carry various proteins, enzymes, miRNAs, bile acids, and lipids, which all have a potential functional role. HDLs are dynamic particles, being either protective or deleterious agents in health or disease. Today's research aims to gain new understanding of HDLs to develop novel therapies and treatments for cardiovascular diseases. This review focuses on the relationship between structure and function as a multifaceted determinant of the complexity of HDLs. Moreover, attention is paid to future perspectives about HDL as a potential vehicle for drug delivery and therapeutic agent against cardiovascular atherosclerotic disease.

### HDLs' Lifecycle

The backbone of HDL is apolipoprotein A1 (apoA1), which is synthesized via forkhead box protein A3 (15) in the liver and in the intestine. Then ApoA1 is lipidated by ABCA1-mediated cholesterol efflux to form nascent discoidal pre-β HDLs. Lipidation, as well as the conversion of free cholesterol to cholesterol esters, drives the formation of mature spherical α-HDL (16). Mature HDL undergoes constant dynamic remodeling in its 4 to 5 day lifecycle through interactions with a variety of enzymes, such as hepatic and endothelial lipase, generating smaller subspecies (e.g., pre-β HDL) from larger ones (e.g., α-HDL) (16). The dynamic remodeling of HDLs can now be visualized *in-vivo* using fluorescent probes (17). Of note, HDLs play an important role in uptake from the gut and transport into the systemic circulation of antioxidants, such as carotenoids and vitamins of dietary origin (18). Indeed, HDL structure and lipidome are modified post-prandially and in relation to the magnitude of post-prandial triglyceridemia HDL may acquire larger size and a triglyceride rich phenotype (19). Along this evidence, it has been suggested that non-fasting HDL concentrations may be more appropriate predictors of cardiovascular events than fasting levels (20, 21). The underlying biological explanation is still unclear as for instance, a major HDL antioxidant enzyme, paraoxonase-1 (PON-1) activity may not decrease along the postprandial stage (19). The post-prandial metabolism of HDL is still poorly detailed and would benefit from additional investigations in larger groups of individuals in health and disease conditions.

As previously mentioned, the main function of HDL is to scavenge excess cholesterol through RCT and shuttle it to the liver, to organs with high-cholesterol requirements or exchange it with apoB particles (e.g., LDL) (16) for disposal. HDLs deliver cholesterol to the liver and steroidogenic tissues through binding its receptor scavenger receptor B1 (SR-B1), which functions in stable multimers in the plasma membrane for binding HDLs (22). HDLs also interact with ATP-dependent transmembrane transporter proteins, ABCA1 and ABCG1 (23) expressed in macrophages, adipose tissue, gut and liver at high levels (24, 25) for cholesterol delivery.

HDL holoparticles are endocytosed into their target cell types by CD36 and potentially SR-B1 (26, 27), where they may accumulate in the cell or be rapidly retro-endocytosed through yet unknown mechanisms (28). HDLs also enter target cells through micropinocytosis in the lymphatic system (29) or via clathrin-coated pits in a receptor-independent manner in endothelial cells (30). Finally, HDLs undergo transcytosis through polarized cells, mediated by SR-B1 in hepatocytes and interactions between SR-B1 and vascular endothelial growth factor receptor 2 (VEGFR2) in endothelial cells (31).

The liver is the major organ responsible for HDL clearance through the canonical ecto-F_1_-ATPase/PY2_13_ pathway, wherein upregulation of its components increases HDL clearance from the circulation (16). De-lipidated apoA1-particles are cleared, preferentially by the kidneys, through selective SR-B1 uptake (16). Recent evidence suggests that HDLs can integrate into the lipid bilayer of cells (32). Whether this mechanism is permanent or transient is unknown, but it could prove to be a novel method of HDL clearance. Figure 1A summarizes the HDL lifecycle in its key components.

### HDLs Structural Diversity

HDLs are complex particles, which can be separated into several subclasses based on their differing physicochemical properties (33). There is no consensus regarding the definitive categories of HDL subclasses or exactly how to define them, which, combined with the various methods of HDL isolation (33) (Supplementary Table 1), hampers our understanding and ability to investigate HDL biology and role in vascular and metabolic disease. However, considerable efforts were made to classify HDLs in a systematic way. Experts ranging from basic science to clinical practice have devised a five-part sub-classification for HDLs, which encompasses all aforementioned properties: very large HDL, large HDL, medium HDL, small HDL and very small HDL (34). Although it has not replaced the previously detailed, heterogenous classification system, it can be a useful clinical tool. The function and metabolism of HDLs can be influenced by the subclass it belongs to (33, 35), and the ability to distinguish between HDL-subclasses may be both clinically relevant (36) and a reason for statin-therapy success (37). With new gold-standard techniques of classification, such as nuclear magnetic resonance (NMR) (38) which has the advantage of measuring HDL classes from whole plasma without preliminary isolation, major efforts are now focusing on elucidating the complex lipidome (39), proteome (40), and structural subtleties of HDL particles and subclasses (41). We recommend that further clinical studies should establish reference values for the technique adopted, in particular NMR, and should assess whether integration of HDL subclasses measurements and parameters of HDL functionality with patient-specific biomarkers can enhance the stratification of patients for differential diagnosis, disease progression and responses to therapy.

## HDLs are an Important Player in Health and Disease

### HDL Function in Healthy Conditions

The diverse protein and lipid composition of HDL contribute to its atheroprotective function (41). In the vessel wall, HDL undergoes transcytosis through endothelial cells into the sub-endothelial space, where it can efflux cholesterol from foam cells (cholesterol-loaded macrophages), preventing plaque formation. Receptors mediating RCT vary between HDL subtypes, with small pre-β HDL having greater affinity for ABCA1-dependent cholesterol export and α-HDL for ABCG1 (42). Beyond RCT, HDLs have several other beneficial properties, such as anti-oxidant capacity, nitric oxide (NO) production stimulation, anti-inflammatory (i.e., anti-vascular adhesion molecule-1 expression) and anti-apoptotic actions (43). One of the most important properties of HDL is its ability to induce NO-production in endothelial cells, through activation of surface receptors, such as SR-B1 (44) and S1P3R (45), and intracellular signaling cascades, involving Akt, PI3K, and MAPK (46), converging, in-part, on endothelial nitric oxide synthase (eNOS). HDL may also act to stabilize eNOS away from catabolism (47). In atherosclerotic coronary artery disease patients, larger HDL particles have a less anti-oxidative capacity than smaller, denser ones (48), which could be explained by an altered proteome. Larger HDL particles are correlated to apolipoprotein A2, which has been shown to decrease the association between HDL and PON-1, an HDL-bound detoxifying enzyme, by displacing it in a broadly concentration-dependent manner (49). Further, small, dense HDL3 have a more potent anti-inflammatory effect than larger HDL2, demonstrated by their highly effective ability to inhibit TNF-α induced VCAM-1 expression in an *in-vitro* endothelial cell model. Here, proteomic modifications were not responsible, as the artificial substitution of apolipoprotein 1 by apolipoprotein 2 in HDL3 did not alter the beneficial anti-inflammatory profile (50). Interestingly, increasing evidence seems to point to a disease-specific HDL-size function relationship, while smaller HDLs seem to protect against atherosclerosis (51), in dysmetabolic diseases, like Type 2 Diabetes Mellitus (T2DM), larger HDLs seem beneficial (52), potentially due to improved RCT function or a different molecular composition.

The lipidome of HDL has been demonstrated to have functional properties (39). In healthy conditions, phospholipids (PL) are the dominant HDL lipid component (up to 50% of HDL lipids) and seem to stabilize the particle (53). A composition shift toward phosphatidylcholine promotes cholesterol efflux, while an increase in sphingomyelin decreases influx of cholesterol via SR-B1 (54). Most recently, the sphingosine-1-phosphate receptors (S1PR) have garnered increasing attention as an HDL target receptor, since 50 to 70% of plasmatic S1P is carried by HDL particles. The activation of S1PR1 and S1PR3 by HDL has protective effects on endothelial cells, reducing inflammation and apoptosis (55). Specifically, S1P enrichment of HDL inhibits oxidized low-density lipoprotein induced apoptosis and increases NO production (56). *In-vitro*, apolipoprotein M, a component of HDL, seems to facilitate the interaction between S1P-HDL and its receptor (57). Others report that there is crosstalk between SR-B1 and S1PR following activation by HDL particles, which would potentiate signaling efficiency. Finally, HDLs are an effective carrier of circulating microRNA (miRNA) to target cells (58), with miRNA potentially being important in stabilizing HDL (59). The miRNA function of miR-223 and miR-24 are best characterized, with miR-223 conferring a beneficial anti-inflammatory profile (60), while miR-24 may be atherogenic (61). As with HDL function, proteome and lipidome composition, the miRNA profile of HDL is altered in pathological conditions (62).

### HDL Dysfunction in a Pathophysiological State

Disease states can cause HDL dysfunction as visualized in Figure 1B. In 2011, Besler et al. showed that HDLs isolated from patients with chronic coronary disease and acute coronary syndrome were significantly less able to stimulate NO production *in-vitro*, and exerted pro-oxidative and pro-inflammatory actions (43). Recently, the strong association with acute coronary syndrome (63) has been further extended to low cholesterol efflux capacity values and low HDL levels of S1P and apoA1.

In chronic kidney disease, the increased association of symmetric dimethylarginine to HDL alters HDL functionality and directly leads to the development of cardiovascular disease, as it impairs HDL RCT capacity and decreases its anti-inflammatory properties (64). HDLs from patients with valvular heart disease, including rheumatic heart disease, HDLs are pro-inflammatory and uncouple eNOS, which in turn impairs endothelial ability to produce NO (65). Similarly, HDLs from patients with T2DM impair NO production and are pro-inflammatory (66). Alterations in the lipidome, such as increase in triglycerides or decrease in phospholipids (67, 68), or a concomitant increase in surface rigidity due to an altered sphingomyelin to cholesterol ratio, reduce the RCT ability of HDLs, and its ability to associate to beneficial enzymes and proteins (69). Recent studies suggest that HDL-triglycerides measurement may be a useful biomarker to determine HDL quality and HDL function over HDL-C (70). While it has been widely accepted that oxidation and glycation of HDLs are a major driver of HDL dysfunction *in-vivo* (71, 72), a few studies challenge this view, finding either no dysfunction (73) or improved function (24, 74) following either endogenous or artificial oxidation of HDL.

Lipid composition, size and structure of HDLs are closely linked. In T2DM patients, several studies show that there is a shift toward smaller HDL particles, and an increase in triglyceride presence on HDL (75), which may render them more hydrophobic and therefore challenging the idea that small HDL is always protective, but rather suggesting a close interplay between HDL size-composition-function and each specific disease condition. HDLs are direct players of whole-body glucose homeostasis (76), through activating AMPK-dependent glucose uptake (77), increasing insulin secretion (78), and protecting pancreatic ß-cells from apoptosis (79). Thus, T2DM may influence HDL function, and HDL function may in turn influence T2DM pathogenesis. However, to date we do not yet have clear evidence about the functional consequences of all structural alterations, which may contribute to the dysfunction of HDLs in T2DM. Moreover, macrophage-associated enzyme myeloperoxidase, which is increased in atherosclerotic cardiovascular disease, can catalyze deleterious changes to HDL associated proteins, namely apoA1, causing an impaired RCT ability and increase in inflammatory pathways (51). Serum amyloid A is a causal factor of HDL dysfunction, inducing a loss of anti-inflammatory and RCT function and a decreased ability of HDLs to interact with the plasma membrane of adipocytes (80). Beyond the above described roles of HDL, there are additional key roles of HDL in immunity (81, 82), Alzheimer's prevention (83), and even cancer survival (84) as mentioned in Table 1, which could not be covered in this mini-review.

**Table 1:** HDL as a therapeutic tool.

	**Disease studied**	**Method**	**HDL-intervention**	**Conclusions**	**PMID**
Cardio-metabolic Diseases	Acute Coronary Syndrome	Human	Autologous delipidated serum diffusion	Well-tolerated in patients with ACS	20538165
		Human	CER-001	Treatment did not reduce coronary atherosclerosis	24780501
		Human	CSL112	Repeated infusions were safe and well-tolerated	24122814
	Coronary Artery Disease	Human	MDCO-216	↑ atherogenic lipid profile (unexpected) (27816804), ↑ apoA1, ↑ phospholipids, ↑ pre-β HDL, at high doses (>20 mg/mL) ↑ TG, ↓ HDL-C (27418968)	27816804, 27418968
		Human	CSL112	↑ apoA1, ↑ cholesterol efflux, ↑ pre-β HDL	24969776
		Mouse and human	HDL-CAD loaded with S1P	Restored HDL function (vasodilatation in *ex-vivo* myograph mouse aorta), restored ERK and Akt signaling	26403344
	Myocardial Ischemia	Rat	rHDL VEGF	Efficient delivery of VEGF, 13% ↑ of ejection fraction over controls	Sun et al. (85)
	Type 2 Diabetes Mellitus	Human	Extended release niacin therapy	↑ improves HDL vaso-protective properties, ↓ oxidation and ↑ NO production	20026785
		Human	Pioglitazone administration	↓ oxHDL, HDL-C remain constant (30740640), ↓HDL-T (25137425)	30740640, 25137425
		Human	RVX-208	Δ HDL lipidome, HDL-C remain constant	27173469
		Mouse	HDL infusion	↓ plasma glucose, ↓ inflammation, ↑ muscle glycogen, ↑ pancreatic islet structure (23166092), ↑ glycemic control, ↑ insulin sensitivity, ↑ glucose uptake into muscle, ↑ glucose disposal, ↑ glucose phosphorylation (27193916)	23166092, 27193916
		Mouse	MDCO-216	Reversed CV dysfunction and heart failure in T2DM-induced by HSHF diet	30871282
		Human	rHDL infusion	↓ fasting lipolysis, ↓ FA oxidation, ↓ circulating glycerol, ↑ NEFA (21224289), ↑ Cholesterol Efflux, ↑ Anti-inflammatory properties (19281927)	21224289, 19281927
	Atherosclerosis	Rabbit and human, *in-vitro* HCAEC	ETC-642	Anti-inflammatory effects via inhibiting TNF-α, VCAM-1 ICAM-1, no change in HDL lipid composition (22128776), Anti-inflammatory comparable to native ApoA1, via NFκB inhibition (21571275), Phase-I Clinical Trial showed it was safe and well-tolerated in humans in a range of doses (86)	22128776, 21571275, Khan et al. (86)
		Rabbit and human	ETC-216	6% ↓ soft plaques with ETC-216, 5% ↓with apoA1 Milano and plaque unchanged in placebo group, ↓ macrophage density at plaque (18342230), in humans ↓ mean atheroma volume by 1.06% (14600188)	18342230, 14600188
		Human and mouse	CSL111	↑ hApoA1, ↑ hpre-β HDL, ↑ total cholesterol, ↑ TG (22067613), ↓ mean atheroma volume by 3.4%, treatment group had abnormal liver function (17387133)	22067613, 17387133
		Rabbit and human	CSL112	↑ HDL-VS, ↑ efflux capacity in treated compared to native HDL, ↑ ABCA1 dependent efflux	23868939
		Human and mouse	CER-001	↑ cholesterol elimination, ↓ inflammation, ↓ plaque size, ↓ lipid content of the plaque, 80% ↓ macrophage in plaque (24401224), CHI-SQUARE trial: treatment did not reduce coronary atherosclerosis (24780501), CARAT trial: no reduction of atherosclerotic plaques, no change in plaque composition (28567351)	24401224, 24780501, 28567351
		Human	rHDL infusion	↓ VCAM-1, ↓ plaque lipids, ↓ macrophage size, ↑ HDL-C	18832751
		Mouse	ELK-2A2K2E	↑ Cholesterol Efflux, ↓ Atherosclerosis, ↓ Vascular Inflammation and Oxidation	23874769
		Mouse	4F	↓ early atherosclerosis lesions, ↓ inflammation, no change in mature atherosclerotic lesions	20876212
		Mouse and rabbit	ApoE mimetics	↑ HDL PON-1 activity, ↓ atherosclerosic lesions, ↓ inflammation	20221865
		Mouse, rabbit human cell-lines	rHDL loaded with anti-atherosclerosis drugs	Statin: ↓ inflammation in advances plaques, inhibits progression of inflammation (24445279),	24445279, 23069716,
				Tanshinone IIA: ↑ anti-atherogenic capacity than drug alone (23069716, 21835236), Atorvastatin and dextran sulfate coat: ↑ delivery of drug to macrophages, ↓ oxLDL uptake (28004910), Lovastatin: Inhibition of oxLDL internalization and ↓ of 50% of intracellular lipid load compared to lovastatin alone (29382194), Simvastatin: ↓ macrophage proliferation, ↓ plaque inflammation, favorable plaque remodeling (26295063), Statins and Hyaluronic Acid (HA) encapsulation: HA encapsulation resulted in ↑ uptake in atherosclerotic plaques, ↓ uptake in the liver (24947229, 28144137) and ↓ inflammation (29885417)	21835236, 28004910, 29382194, 26295063, 24947229, 28144137, 29885417
		Mouse	rHDL loaded with tracer agent	Can be used to detect atherosclerotic lesions (12007282), Gd-based agent allowed for more effective contrast imaging of atherosclerotic plaques (19378935), the use of oxidized ApoA1 improved the uptake in macrophages significantly (24729189), Fe-O-based contrast agent allows specific imaging of cellular and sub-cellular locations of HDL localization (20926130), P2fA2: Effective imaging of atherosclerotic plaques in MRI (19072768)	12007282, 19378935, 24729189, 20926130, 19072768
Other diseases	Alzheimer's disease	Mouse, SAMP8	ApoE3-rHDL, ApoJ-rHDL	rHDL passes the blood-brain barrier and accelerates Aβ clearance (24527692), accumulation in the cranial region (29116115)	24527692, 29116115
	Cancer	Mouse and human	rHDL with paclitaxel	↑ cytotoxicity in cancer cell lines than drug alone, ↑ tolerance *in-vivo* than drug alone (18176115), No drug leakage or remodeling of rHDL, efficient delivery to tumor (24079327), 30% increase uptake into cancer cells than drug alone (19637935)	18176115, 24079327, 19637935
		Mouse and human	rHDL loaded with siRNA	Effective delivery to cancer cells via SR-B1(28717350), VEGF siRNA: ↓ VEGF expression levels, ↓ tumor angiogenesis, ↓ intratumoral microvessels (24875759), Effective co-delivery to cancer cell lines over-expressing SR-B1 (28753317)	28717350, 24875759, 28753317
		Mouse and human	rHDL loaded with imaging agents	Imaging and monitoring of tumor associated macrophages more efficient than (89)Zr-rHDL imaging agent alone (26112022), rHDL labeled with 99mTc and hydrazinonicotinic acid is an effective new radio-tracer for labeling tumors (30543234), apoE3 rHDL-AuNP results in effective labeling of LDLR overexpressing cancer cell lines (29225464)	26112022, 30543234, 29225464
		Mouse and human	rHDL loaded with anti-cancer drugs	PTX-HZ08-rHDL NPs target tumors via SR-B1, ↓ drug leakage, ↑ anti-tumor capacity than drug alone (27343697), Triple-negative breast cancer cells better targeted and less off target effects observed in cardiomyocytes (rHDL with apatinib and valrubicin) (28670138), 100-fold improvement in selective therapeutic efficiency (rHDL with fenretinide) (24459664), ↑ anti-tumor response compared to free drug cocktail, ↑ anti-cancer effects, ↑*in-vitro* cell toxicity (rHDL with paclitaxel and doxorubicin) (27982602), Effective receptor mediated uptake, overcomes solubility barrier of AD-32 [rHDL with valrubicin (AD-32)] (22393294)	27343697, 28670138, 24459664, 27982602, 22393294
		Human, clinical trial Phase 1	rHDL loaded with miRNA (MRX34)	Safe, well-tolerated, preliminary evidence of anti-tumor activity	27917453
		Mouse	HDL-NP, gold nanoparticle conjugated	Selectively promotes cholesterol efflux, not cholesterol delivery, to lymphoma cells, resulting in cell starvation and apoptosis	23345442

*Overview of pre-clinical and clinical research, of the last 10-years focusing on HDL. Several excellent reviews exist for further reading (87–90)*.

### The Paradox of Extremely High HDL

Recent data points to high levels of HDL-C as potentially deleterious to cardio-vascular health, showing a distinct U-shape association between HDL-C above 100 mg.dL^−1^ and disease risk (91, 92) in men. Raised HDL-C may increase disease risk for several reasons, including potential undisclosed confounders. Genetic mutations causing elevated HDL may also be a risk factor for disease, a potential reverse causation arising from the severity of disease in the studied at-risk population, or the possibility that HDLs becomes dysfunctional at such elevated circulating levels. The cut-off for pathologically high HDL is not clearly defined, but has tentatively been placed as HDL-C levels ranging from 60 to 80 mg.dL^−1^. A recent cross-sectional study determined that in men, and after adjusting for cardiovascular risk factors, extremely high HDL-C was associated to endothelial dysfunction, as measured *in-vivo* by flow mediated vasodilation (FMD), while low HDL-C was not (93). Less than 10% (93) of the population present extremely high HDL-C levels, but this feature is more frequent in Type 1 Diabetes Mellitus (T1DM) (94). HDLs isolated from young T1DM patients are dysfunctional, less able to induce NO production by endothelial cells and pro-oxidant. Further, T1DM patients with extremely high HDL levels and inflammation have a substantially decreased FMD (94), suggesting that high levels of HDL associated to systemic inflammation, as found in several cardiovascular and metabolic disease, may be a driver of vascular dysfunction and not merely a reflection of an overall pathological state.

## HDLs: Therapeutic Avenues

### HDLs Recover After Metabolic Interventions

Recovery from metabolic and cardiovascular disease parallels restored HDL functionality and increased HDL concentration (95). Roux-en-Y gastric bypass (RYGB) is a bariatric surgery able to decrease cardio-vascular mortality (96) and resolve T2DM in a rapid and body weight-independent manner (97). We have demonstrated in both humans and rodent models that RYGB promotes an early improvement of HDL function, including cholesterol efflux capacity, anti-apoptotic, anti-oxidant and anti-inflammatory activity, and increased capacity to produce NO (98). BMI-matched controls to the 12 week post-surgery patient group did show impaired HDL function, demonstrating that post-surgical improvements in HDL function occurred in a body weight-independent manner (98). Evidence from follow-up studies indicates that the restoration of HDL function is stable long term after bariatric surgery. Interestingly, evidence shows that HDLs tend to be larger post-RYGB, further increasing the complexity of HDL-size-composition-function relationship discussed above (99).

Exercise and diet also improve HDL function. In chronic heart failure patients, a 15 week exercise intervention significantly improved the ability of HDLs to activate eNOS and produce NO (100). One study shows that HDLs isolated before and after an exercise-based weight loss intervention showed significant correlation between RCT and amount of weight lost (101), and HDL levels significantly increase post-exercise training across different studies (101–103). While RYGB seems to acts via additional mechanisms (Figure 1B) (98), body weight loss has beneficial effects on HDLs, leading for instance to increased HDL2 particle number after dieting (103), to improved efflux-capacity (104) and altered miR223 expression (105). Further, increases in brown fat metabolism, which is impaired in obese subjects, correlates to beneficial HDL remodeling, in both humans and mouse models (106).

### HDL-Based Therapies

Manipulation of HDL components have beneficial effects. Enrichment of S1P to reconstituted HDL (rHDL) induce better vasorelaxation than control rHDL (107). In humans, a small trial found that short-term infusion (4 weeks, 1 infusion per week) of rHDL was able to significantly decrease endothelial progenitor cell (EPC) apoptosis in patients with acute coronary dysfunction, and increase the circulating chemokine levels known to be important in EPC recruitment, such as stromal cell-derived factor-1 or vascular endothelial growth factor (108). Another small-scale human trial found that rHDL infusion resulted in decreased plaque lipid content and decreased expression of VCAM-1 on the plaque surface (109). Preliminary results from a larger clinical trial found that while plaque size *per se* had not regressed following rHDL infusion, there was a significant improvement in the plaque characterization index and overall coronary score (110). Furthermore, increasing apoA1 levels alone, either through genetic manipulation in animal models (111) or through exogenous infusion in animals (112) and humans (113), was enough to provide beneficial effects on atherosclerotic plaque regression (114). For certain parameters HDL still outperformed apoA1 mimetic alone (115). Animal studies show that raising an apoA1 and functional HDL can promote atherosclerosis plaque regression through inhibition of inflammation and decreased activation of immune cells (116). Larger trials paint a more controversial story, showing no detectable effect after supplementation of an engineered pre-β HDL mimetic on atherosclerotic plaque composition or regression compared to placebo (117).

### HDLs for Drug Delivery

For over 10 years, rHDLs have been used in research for treatment delivery (118). The delivery to organs of interest is efficient and the cargo is protected from degradation. While conjugations of HDLs have mostly been used to target the liver, where SR-B1 expression is high, it has been found that the addition of folic acid to HDLs expands the target organ pool to cells expressing the folate receptor (119). The current understanding of how to encapsulate vaso-protective compounds within rHDL allows us to consider using it a treatment (120). The infusion of rHDL loaded with a potent LXR agonist enabled atherosclerotic plaque regression in the apoE-knock out mouse model, with significant accumulation of the synthetic HDL found in the atherosclerotic lesions (121). Similarly, rHDL encapsulating statins (S-rHDL) are more effective at reducing atherosclerosis-induced inflammation than statin or rHDL alone in mice (122). Moreover, rHDLs have been used for contrast imaging in MRI (123). Predictions around the future developments in rHDL-based therapies evolve around developing rHDL particles that act simultaneously as drug delivery and imaging systems, termed “theranostics” (120) included in Table 1.

## Conclusion: HDL is an Incompletely Understood, Complex, and Dynamic Particle With Therapeutic Potential

Today, HDLs are considered as multifaceted entities beyond their cholesterol-carrying action. We attempt to understand the multiple HDL functions and the responsible mechanisms. Indeed, we are now moving away from the dualistic model of “good” and “bad” cholesterol and are constructing a more complex and realistic image of HDLs, including identifying various subclasses of HDLs using new techniques, and defining the proteome and lipidome of different HDL subclasses in health, disease and after therapies. In this review, we report new evidence about changes in size and composition as determinants of functionality. Further, the emergence of data from patients with ultra-high HDL levels challenges our understanding of HDL roles and functions. While clinical results on HDL-based therapies remain controversial, a more refined understanding of HDLs can lead to design more efficient clinical treatments involving these complex particles. Similarly, HDLs potential as therapeutics, although promising, is contingent on further research.

## Author Contributions

AJ and EO wrote the manuscript and figures. EO conceived the manuscript.

### Conflict of Interest

The authors declare that the research was conducted in the absence of any commercial or financial relationships that could be construed as a potential conflict of interest.

## Supplementary Material

Supplementary Table 1 Details of the different HDL sub-classification categories based on the different methods of HDL separation. Nuclear magnetic resonance (NMR) [method described in Otvos et al. (38)] has the advantage of measuring HDL classes from whole plasma and thus does not require preliminary isolation.

## Abbreviations

ABCA1: ATP-Binding Cassette Transporter 1
apoA1: Apolipoprotein A1
ApoA2: Apolipoprotein A2
CD36: Cluster of Differentiation 36
CETP(i): Cholesteryl Ester Transfer Protein (inhibitor)
eNOS: Endothelial Nitric Oxide Synthase
EPC: Endothelial Progenitor Cells
FMD: Flow Mediated Vasodilation
HDL: High Density Lipoproteins
HDL-C: HDL cholesterol
HDL-TG: HDL triglycerides
LDL: Low Density Lipoprotein
miRNA: Micro Ribonucleic Acid
NO: Nitric Oxide
NMR: nuclear magnetic resonance
PL: Phospholipid
PON-1: Paraoxonase-1
RCT: Reverse Cholesterol Transport
rHDL: Reconstituted HDL
RYGB: Roux-en-Y Gastric Bypass
S1P: Sphingosine-1-Phosphate
S1PR: Sphingosine-1-Phosphate Receptor
SM: Sphingomyelin
S-rHDL: Reconstituted HDL encapsulated statin
T2DM: Type 2 Diabetes Mellitus
TNF-α: Tumor Necrosis Factor α
TG: Triglycerides
VEGF-A: Vascular Endothelial Growth Factor A
VEGFR2: Vascular Endothelial Growth Factor Receptor 2
VLDL: Very Low-Density Lipoproteins
